# Urokinase Plasminogen Activator Receptor: An Important Focal Player in Chronic Subdural Hematoma?

**DOI:** 10.1007/s10753-023-01957-5

**Published:** 2024-01-18

**Authors:** Thorbjørn Søren Rønn Jensen, Markus Harboe Olsen, Giedrius Lelkaitis, Andreas Kjaer, Tina Binderup, Kåre Fugleholm

**Affiliations:** 1 Department of Neurosurgery, The Neuroscience Center, Copenhagen University Hospital, Inge Lehmanns Vej 6, 2100 Rigshospitalet, Copenhagen, Denmark; 2 Department of Neuroanesthesiology, The Neuroscience Center, Copenhagen University Hospital, Rigshospitalet, Copenhagen, Denmark; 3 Department of Anaesthesiology, Zealand University Hospital, Køge, Denmark; 4 Department of Pathology, Rigshospitalet, Copenhagen, Denmark; 5 Department of Clinical Physiology, Nuclear Medicine and PET & Cluster for Molecular Imaging, Copenhagen University Hospital-Rigshospitalet & Department of Biomedical Sciences, University of Copenhagen, Blegdamsvej 9, 2100 Copenhagen, Denmark

**Keywords:** urokinase plasminogen activator surface receptor, chronic subdural hematoma, recurrence, recurrent CSDH, pathophysiology, histology, luminex, H-score.

## Abstract

Chronic subdural hematoma (CSDH) development involves inflammatory, angiogenetic, and fibrinolytic mechanisms, several components of which are now unraveled through intensive research. The urokinase plasminogen activator receptor (uPAR) is part of the plasminogen activator system and possesses inflammatory, angiogenetic, and fibrinolytic capabilities. As a first, this study aims to identify uPAR in the hematoma fluid, hematoma membrane, dura mater, and systemic blood from patients with CSDH and, if present, to investigate if the uPAR level at the time of surgery may be a predictor for later developing recurrent CSDH. uPAR expression in the hematoma membrane and dura mater was analyzed using immunohistochemistry and presented as the H-score of the positive immunostaining. The uPAR levels in the hematoma fluid and systemic blood were determined using a multiplex antibody bead kit (Luminex). Samples were collected at the time of the first CSDH surgery, and in the case of recurrent CSDH within 90 days, the samples were again collected at reoperation. A comparison of uPAR expression between the hematoma membrane and dura mater, as well as uPAR levels in systemic blood and hematoma fluid, was performed using the Wilcoxon rank sum test. We included 112 patients, 26 of whom had recurrent CSDH. The median hematoma uPAR level was 22,125 (14,845–33,237) and significantly higher than the median systemic blood level of 789 pg/L (465–2,088) (*p* < 0.001). Similarly, the uPAR level of the hematoma membrane was 14.3 (7.54–44.8) and significantly higher than the dural uPAR level of 0.81 (0.3–1.98) (*p* < 0.001). For the first time, we identified uPAR in the subdural fluid, hematoma membrane, dura mater, and systemic blood from patients with CSDH. The high expression of uPAR in the subdural fluid and hematoma membrane indicates that the mechanisms of CSDH are predominantly in the subdural fluid collection and surrounding hematoma membrane.

**Supplementary Information:**

The online version contains supplementary material available at 10.1007/s10753-023-01957-5.

## BACKGROUND

It has become increasingly evident that the pathophysiology of chronic subdural hematoma (CSDH) involves three linked processes, inflammation, angiogenesis, and fibrinolysis [1], which is supported by the discovery of subdural fluid biomarkers participating in all three processes. Although the pathophysiological processes of CSDH have been linked to several immunological, angiogenetic, and fibrinolytic mechanisms, the understanding of the pathophysiology underlying both primary and recurrent CSDH needs further clarification.

The urokinase plasminogen activator receptor (uPAR) is part of the plasminogen activator system and has pleiotropic functions in both physiological and pathological processes. uPAR primarily functions as a receptor for urokinase plasminogen activator (uPA) [2], which catalyzes the activation of the universally present plasminogen into plasmin, thereby degrading fibrin [3]. uPAR is one of the earliest mediators of fibrinolysis, both by fibrin degradation and by tissue remodeling caused by the recruitment and activation of monocytes and neutrophils [4]. Furthermore, uPAR expression is strongly activated during inflammation, immune responses, injury, and stress [3]. uPAR is also expressed in a large proportion of human cancers as an effector in oncogenic signaling pathways and has been shown to be a marker of invasiveness and aggressiveness in several cancers [3, 5]. Physiologically, most cells at rest have no uPAR on their cell membrane [6]. However, when activated, the secreted uPAR (suPAR) interacts in an autocrine and paracrine fashion with neutrophils and macrophages, enhancing the invasive and proliferative properties of these cells during inflammation [7]. Finally, uPAR has shown angiogenetic capabilities in both animal models and humans. In wild-type mice, the inhibition of uPAR reduces tumor growth by inhibiting fibroblast-induced angiogenesis [8], and in humans, uPAR can indirectly reduce the availability of vascular endothelial growth factor (VEGF), which is known to be involved in the angiogenetic processes of CSDH [9, 10]. As the capacities of uPAR are similar to the processes involved in CSDH pathophysiology, measuring uPAR in CSDH patients is warranted.

By investigating the role of uPAR in CSDH, we aimed to [1] identify uPAR in the hematoma fluid, hematoma membrane, dura mater, and systemic blood, [2] investigate if the level of uPAR at the time of surgery for first-time CSDH may predict recurrence, and [3] examine if uPAR expression is different between the first and second operations in patients with recurrent CSDH.

We hypothesized that [1] uPAR is present in hematoma fluid, hematoma membrane, dura mater, and systemic blood from CSDH patients due to similar biological features in both CSDH disease and uPAR, [2] uPAR levels predict CSDH recurrence in patients with primary CSDH, as patients later developing a recurrent CSDH presumably hold a higher level of inflammation, angiogenesis and/or fibrinolysis, and [3] uPAR is elevated at the time of the second operation in patients with recurrent CSDH due to an increasing involvement of either of the three pathophysiological processes.

## METHODS

### Study Population

Adult patients (≥ 18 years old) with CSDH diagnosed on computed tomography (CT) or magnetic resonance were randomly included between January 2020 and September 2021 from the Department of Neurosurgery, Copenhagen University Hospital – Rigshospitalet, Copenhagen, Denmark. Exclusion criteria were known head trauma within 14 days of surgery and a previous intracranial operation for conditions other than CSDH. Each hematoma in patients with bilateral hematomas was considered a separate case, also regarding recurrence, as bilateral hematomas do not necessarily have an identical cellular composition. Therefore, unilateral or bilateral recurrent CSDH from bilateral CSDH was regarded as one or two cases, respectively.

Patient characteristics included sex, age, known head trauma, blood thinning treatment, preoperative comorbidity measured by Charlson’s comorbidity index [11], and preoperative symptoms. Specifically, we registered comorbidities with potential inflammatory involvement, as these may affect the level of uPAR. These comorbidities included current smoking [12], current alcohol abuse [13], hypertension [14], diabetes I and II [15], cancer [16], and chronic obstructive pulmonary disease [12]. Radiological variables included midline shift, hematoma volume calculated by the XYZ/2-method [17], localization, and radiological subtype [18]. A recurrence was defined as the need for reoperation due to symptomatic ipsilateral re-accumulation of the CSDH.

The Scientific Ethical Committee of the Capital Region of Denmark (Journal no. H-20051073) approved this study. Consent for inclusion was obtained from either the patient or the next of kin.

### Sample Collection

Hematoma fluid was collected during a burr hole or craniotomy. To avoid the risk of blood contamination from surrounding tissue, the dura mater was left intact following bone opening. After the bone opening, the dura mater was carefully opened, leaving an intact outer hematoma membrane, and a blunt needle on a 10-mL syringe was inserted through the outer membrane. Ten milliliters of hematoma fluid was aspirated and contained in protamine sulfate and ethylenediamine tetraacetic acid (EDTA) containing siliconized vacuum tubes. Patients were excluded if the dura mater was damaged and a leak of subdural fluid was observed before sample collection. Corresponding systemic venous blood was drawn at the time of surgery. All samples were centrifuged at 7000 rpm for 10 min to remove cells and debris, and the supernatants were stored at − 80 C until later analysis.

Following fluid collection, a 5-mm dura mater biopsy was collected with an underlying outer hematoma membrane. The tissue was put aside in 4% neutral buffered formaldehyde immediately after the procedure. Biopsies were subsequently embedded in paraffin, cut in 4 µm slices, and stored at room temperature. Biopsies from the dura mater and hematoma membrane were technical challenges and could not be performed in all patients. Extending the burr hole was not permitted by the Scientific Ethical Committee of the Capital Region of Denmark, resulting in biopsies being collected in a small cranial opening with an often lesser quality that could not be interpreted. In particular, the number of samples from patients with recurrent CSDH was low, as these patients were typically reoperated using the burr hole from the primary operation, where the dura and membrane had already been harvested.

### Luminex

The uPAR levels in both the hematoma and systemic blood were determined with the Luminex multiplex antibody bead kit (ProcartaPlex, Thermo Fisher Scientific, Denmark), according to the manufacturer’s instructions. The samples were measured in simplex and the standard in duplex. Samples with a value above the calibrated standards were reanalyzed with increased dilution until the proper values were obtained. The uPAR values are presented as pg/mL.

### Immunohistochemistry

uPAR staining was performed manually. The uPAR antibody (catalog number GTX100467) was obtained from GeneTex (Irvine, USA). Dilution was 1:500, determined using positive and negative control staining. The specimens were incubated at 60 °C for 60 min before being deparaffinized in Histo-Clear solution, rehydrated in graded ethanol, and submerged in water. The specimens were exposed to heat-induced epitope retrieval with a CC1 buffer for 15 min before staining with the primary antibody. Horseradish peroxidase-conjugated antibody was used as secondary staining and was performed by incubation for 45 min. Envision DAB+ was used to visualize the reaction.

### Assessment of Immunohistochemistry

All immunohistochemical (IHC)-stained specimens were digitally scored using the open-source software QuPath [19]. The dura mater and the hematoma membrane were digitally labeled, and positive and negative cells were identified based on the mean diaminobenzidine (DAB) signal in the cell cytoplasm. Cell expansion was set to 5 μm, and intensity threshold for uPAR was set to 0.2 for weak intensity (+1), 0.4 for moderate intensity (+2), and 0.6 for strong intensity (+3). The H-score was digitally calculated for both the dura mater and hematoma membrane by adding 3× percentage of strongly stained cells, 2× percentage of moderately stained cells, and 1× percentage of weakly stained cells, resulting in a score ranging from 0 to 300 [20].

### Statistics

Patient characteristics were compared between patients without recurrence and patients with recurrence. Evaluation of the normal distribution of the data was carried out by visual inspection. Fisher’s exact test was used for categorical variables; Student’s *t* test was used for normal distributed data; and the Wilcoxon rank sum test was used for non-normal distributed data. Differences between groups were compared using the Wilcoxon rank sum test and presented with median difference together with 95% Hodges-Lehmann confidence intervals. Furthermore, the prognostic value was evaluated using receiver operating characteristics area under the curve (AUC) statistics presented with a 95% confidence interval. The AUC will be interpreted as representing “no better than chance” (~ 0.5), low accuracy (0.5–0.7), moderate (0.70.9), and high accuracy (> 0.9) [21]. In the analysis of the prognostic value of patients with bilateral CSDH, systemic blood was analyzed per hematoma case and not per patient case. Therefore, the systemic blood for a patient with bilateral CSDH was counted for both the right and left sides. As bilateral hematomas may recur on just one side, counting systemic blood per patient would result in systemic blood being analyzed in both groups. Consequently, the chosen method seems plausible. How patients with bilateral hematomas are statistically managed is a common challenge in CSDH research; however, it is often not often addressed [22]. Results are presented as median H-score (interquartile range (IQR)) for the IHC analyses and pg/L (IQR) for the Luminex analyses.

## RESULTS

### Study Population

**Fig. 1:**
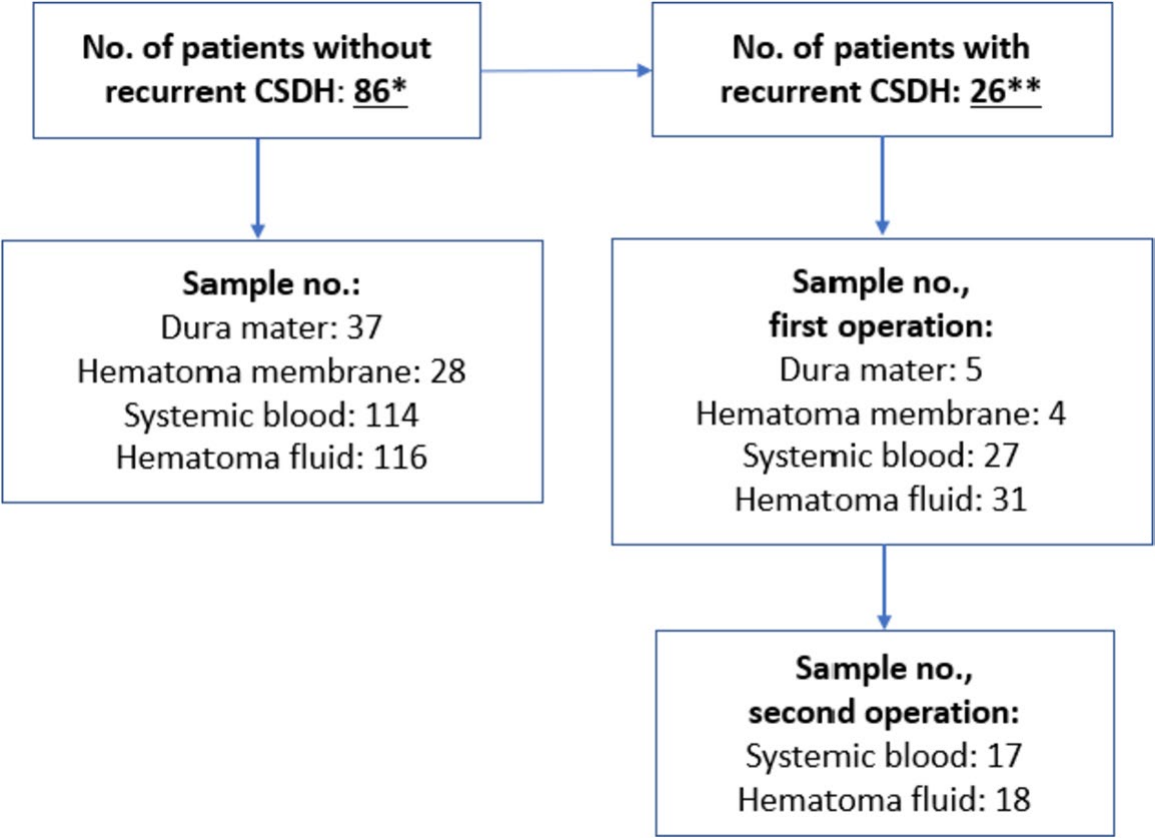
Study samples were drawn from patients with and without recurrent chronic subdural hematoma (CSDH). Samples from the second operation were also drawn from patients with recurrent CSDH. *Of these patients, 21 had bilateral hematomas. **Of these, 15 had bilateral hematomas.

We included 112 patients, 26 of whom had recurrent CSDH within 90 days. Samples were drawn from both the primary and secondary operations, if possible (Fig. 1). When comparing the sex of patients with and without recurrence, only one patient with recurrent CSDH was female (*p* < 0.001). Hematoma localization was different between the two groups, as a higher percentage of patients later developing a recurrent CSDH had bilateral hematomas (*p* < 0.001). Also, the number of patients that died was higher in patients with recurrent CSDH (*p* < 0.001). The remaining patient demographics were not statistically different between patients with and without recurrent CSDH. The full baseline characteristics can be seen in Table 1.

**Table 1:** Patient Demographics with Comparison of Patients With and Without Recurrent Chronic Subdural Hematoma Within 90 Days

	**Patients without recurrence**	**Patients with recurrence**	***p*-value**
No. of patients	86	26	-
**Patients’ demographics**			
Age, median (IQR)	75.5 (19)	77 (13)	0.967
Male sex, *N* (%)	54 (63)	25 (96)	< 0.001*
Performance status before symptom onset, *N* (%)			0.310
0	71 (83)	24 (92)	
1	6	0	
2	8	1 (4)	
3	1	1 (4)	
4	0	0	
**Preoperative status**			
Drugs history, *N* (%)			
Anticoagulant treatment	15	1	0.112
Antithrombotic treatment	20	10	0.137
Both antithrombotic and anticoagulant treatments	0	1	0.232
No anticoagulant or antithrombotic	50	14	0.822
INR, median (IQR)	1 (0.6)	1.1 (0.2)	0.079
Thrombocytes, median (IQR)			
Charlson’s comorbidity index, median (IQR)	4 (3)	4 (2)	0.822
Comorbidity with potential inflammatory involvement, *N* (%)			
Current smoking	16 (19)	1 (4)	0.12
Current alcohol abuse	15 (17)	4 (15)	0.80
Cancer	9 (10)	5 (19)	0.24
Hypertension	38 (44)	11 (42)	0.86
Diabetes I and II	10 (12)	6 (23)	0.14
COPD	6 (7)	0	0.17
Preoperative symptoms			
GCS, median (IQR)	15 (1)	15 (1)	0.597
Headache, *N* (%)	39 (45)	12 (46)	0.942
Vomiting, *N* (%)	5 (6)	3 (12)	0.321
Seizures, *N* (%)	4	1	0.931
Cognitive impairment, *N* (%)	47	15	0.785
Hemiparesis, *N* (%)	52	14	0.548
Aphasia, *N* (%)	15	5	0.835
**Radiological variables**			
Hematoma localization, *N* (%)			< 0.001*
Right	32 (37)	6 (23)	
Left	33 (39)	5 (19)	
Bilateral	21 (24)	15 (58)	
Hematoma volume, mL, median (IQR)			
Unilateral	110.3 (63.3)	103.9 (64.7)	0.907
Bilateral	58.4 (24.2)	65.9 (37.9)	0.368
Midline shift, mm, median (IQR)	7 (8)	8 (8)	0.806
Radiological subtype, *N* (% out of 107/41 hematomas)			0.450
Homogenous	45 (42)	23 (56)	
Separated	3 (3)	2 (5)	
Membranous	29 (27)	8 (19.5)	
Mixed	30 (28)	8 (19.5)	
Outcome			
Death, *N* (%)	6 (7)	4 (15)	< 0.001*

*IQR* interquartile range; *INR* international normalized ratio; *GCS* Glasgow Coma Scale; *COPD* chronic obstructive pulmonary disease

*statistically significant

### Immunohistochemical Staining

uPAR staining showed a marked difference in the number of positive cells between the dura mater and hematoma membrane. Positivity in the dura mater was limited to the cytoplasm of endothelial cells, scattered fibroblasts, and a few perivascular macrophages. There was a slight increase of positive cells toward the side of the hematoma.

**Fig. 2:**
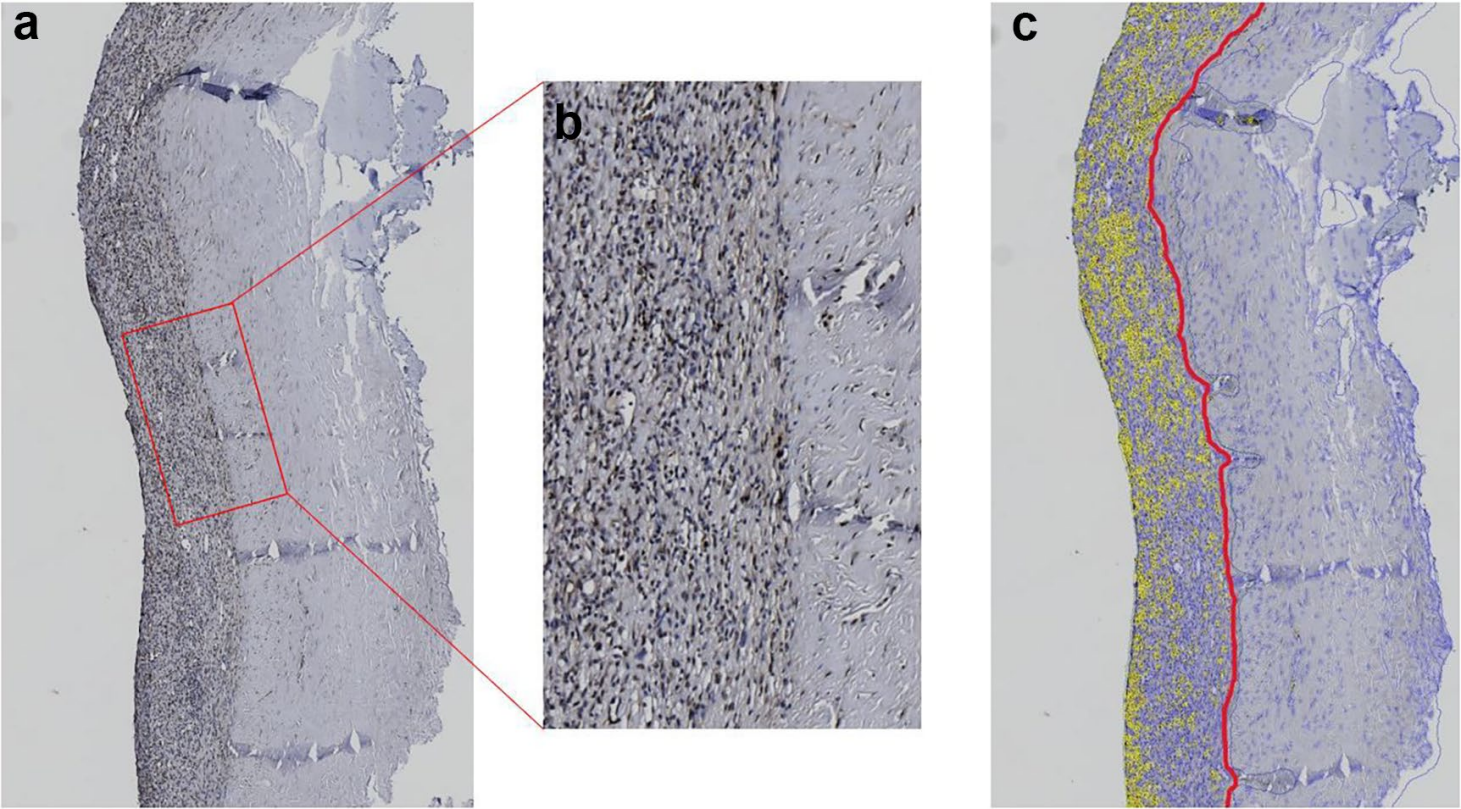
**a** An example of an immunohistochemical sample with the uPAR antibody. uPAR staining demonstrated a clear difference in the number of positive cells between the dura mater and hematoma membrane. **b** Enlargement of the marked area. Positivity in the dura mater was limited to the cytoplasm of endothelial cells, scattered fibroblasts, and a few perivascular macrophages. Positivity in the hematoma membrane was localized in numerous cells with positive, predominantly moderate cytoplasmatic reactions, including macrophages, lymphocytes, fibroblasts, and endothelial cells. **c** Presentation of the boundary between the hematoma membrane and the dura mater (red marking). Cells with positive staining for uPAR have been colored yellow using Qpath [19]. Significantly, more cells within the hematoma membrane were positive for uPAR.

In the hematoma membrane, there were numerous cells with positive, predominantly moderate cytoplasmatic reactions, including macrophages, lymphocytes, fibroblasts, and endothelial cells. Moreover, there was a focal weak positive reaction in the extracellular matrix, which was not included in the digital scoring. Staining in some macrophages may be false positives due to deposits of hemosiderin, which had a similar color due to the chromogen used in IHC staining. Staining intensity was predominantly weak in the dura mater, as well as in the hematoma membrane (Fig. 2).

### Comparison of Systemic and Subdural uPAR Levels

**Fig. 3:**
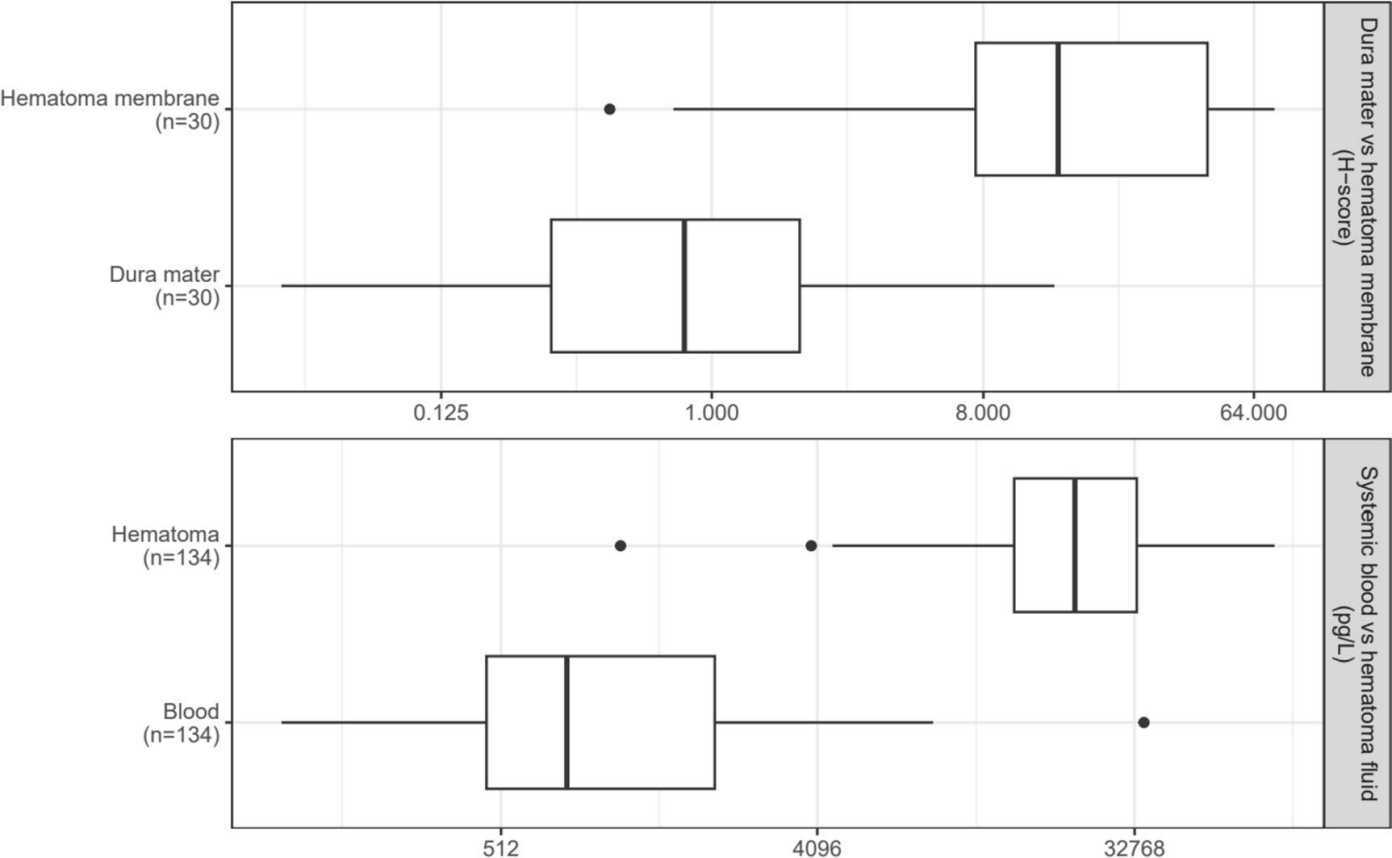
Boxplot presenting comparison of the H-score determined by immunohistochemistry between dura mater and the hematoma membrane (*p* < 0.001) and comparison of uPAR levels between systemic blood and hematoma fluid as measured by Luminex (*p* < 0.001).

To identify a possible role of uPAR in CSDH, we compared uPAR levels between systemic blood and the hematoma fluid using Luminex and uPAR levels between the dura mater and hematoma membrane in IHC analyses (Fig. 2). The IHC analyses demonstrated a median H-score of 14.3 (7.54–44.8) for the hematoma membrane. This was significantly higher than the H-score of the dural uPAR, which was 0.81 (0.3–1.98) (*p* < 0.001). The median hematoma uPAR level was 22,125 pg/L (14,845–33,237) and significantly higher than the median systemic blood level of 789 pg/L (465–2,088) (*p* < 0.001) (Fig. 3).

### uPAR Levels as a Predictor of Recurrent CSDH

To investigate whether uPAR levels measured in patients with primary CSDH could predict the risk of recurrent CSDH, we compared uPAR levels from the dura mater, hematoma membrane, systemic blood, and hematoma fluid between patients with primary CSDH without recurrence and patients who later developed recurrent CSDH.

**Fig. 4:**
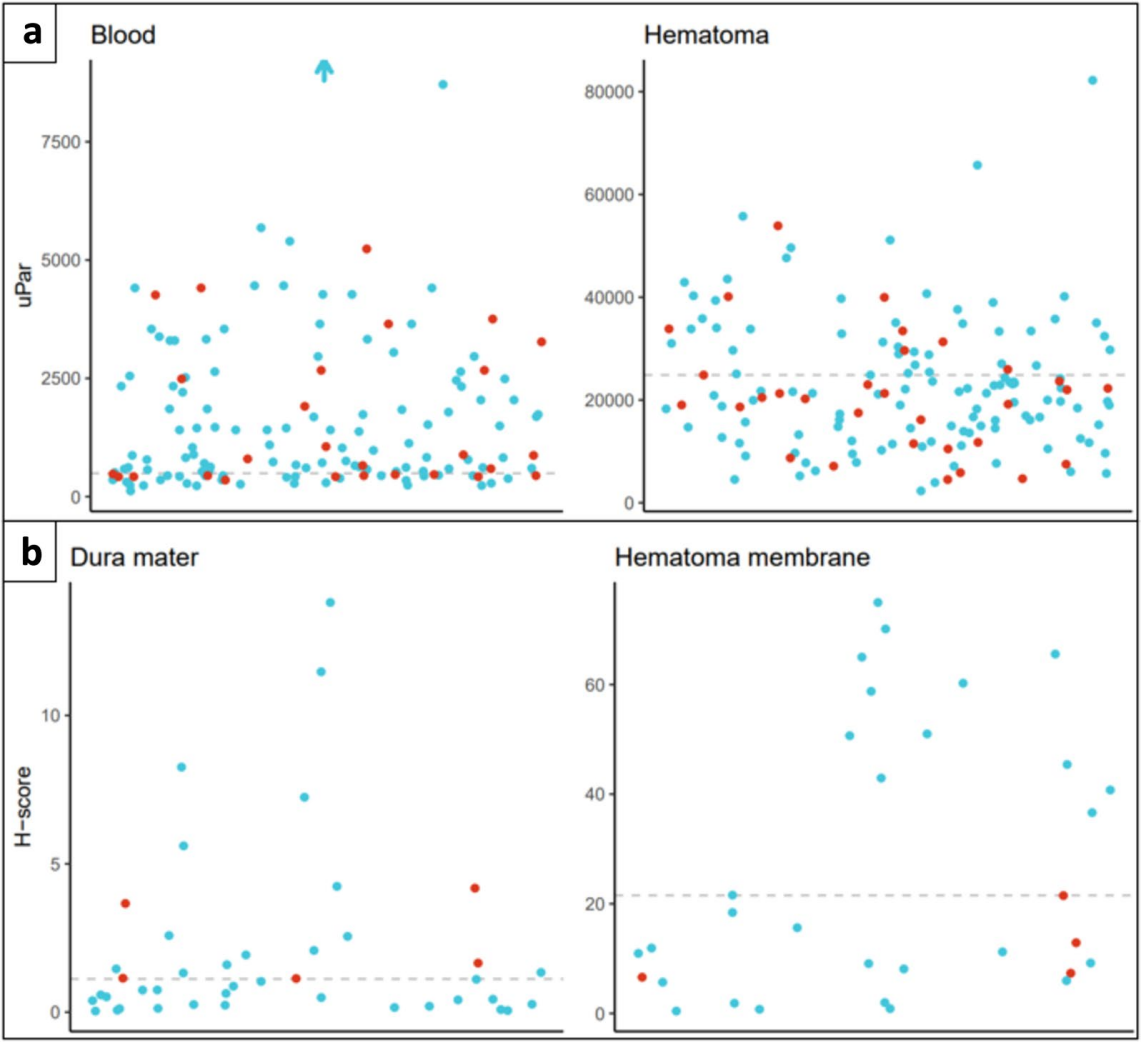
A scatterplot presenting the possible prediction of recurrent CSDH using uPAR levels in the dura mater*,* hematoma membrane, systemic blood, and hematoma fluid by comparing uPAR levels from patients without and with recurrent CSDH within a 90-day follow-up. Levels of uPAR in the dura mater significantly predicted the risk of recurrent CSDH

We found that the AUC of the dura mater was 0.73 (0.57 to 0.89), indicating that the dural uPAR level could predict recurrent CSDH with moderate accuracy. However, the number of recurrent CSDHs in this analysis was 5, and the results should therefore be interpreted with caution. The comparison of uPAR levels in the hematoma membrane, systemic blood, and hematoma fluid could not predict recurrent CSDH (Fig. 4 and Supplementary Table 1).

The number of systemic blood samples is presented per CSDH case and not per patient. Blue dots are cases without recurrent CSDH. Red dots are cases with recurrent CSDH within 90 days. The dotted line represents the median. The scale of the y-axis in the comparison of blood vs. hematoma and dura mater vs. hematoma membranes differs.

### Comparison of uPAR Levels in Recurrent CSDH Between the First and Second Operations

**Fig. 5:**
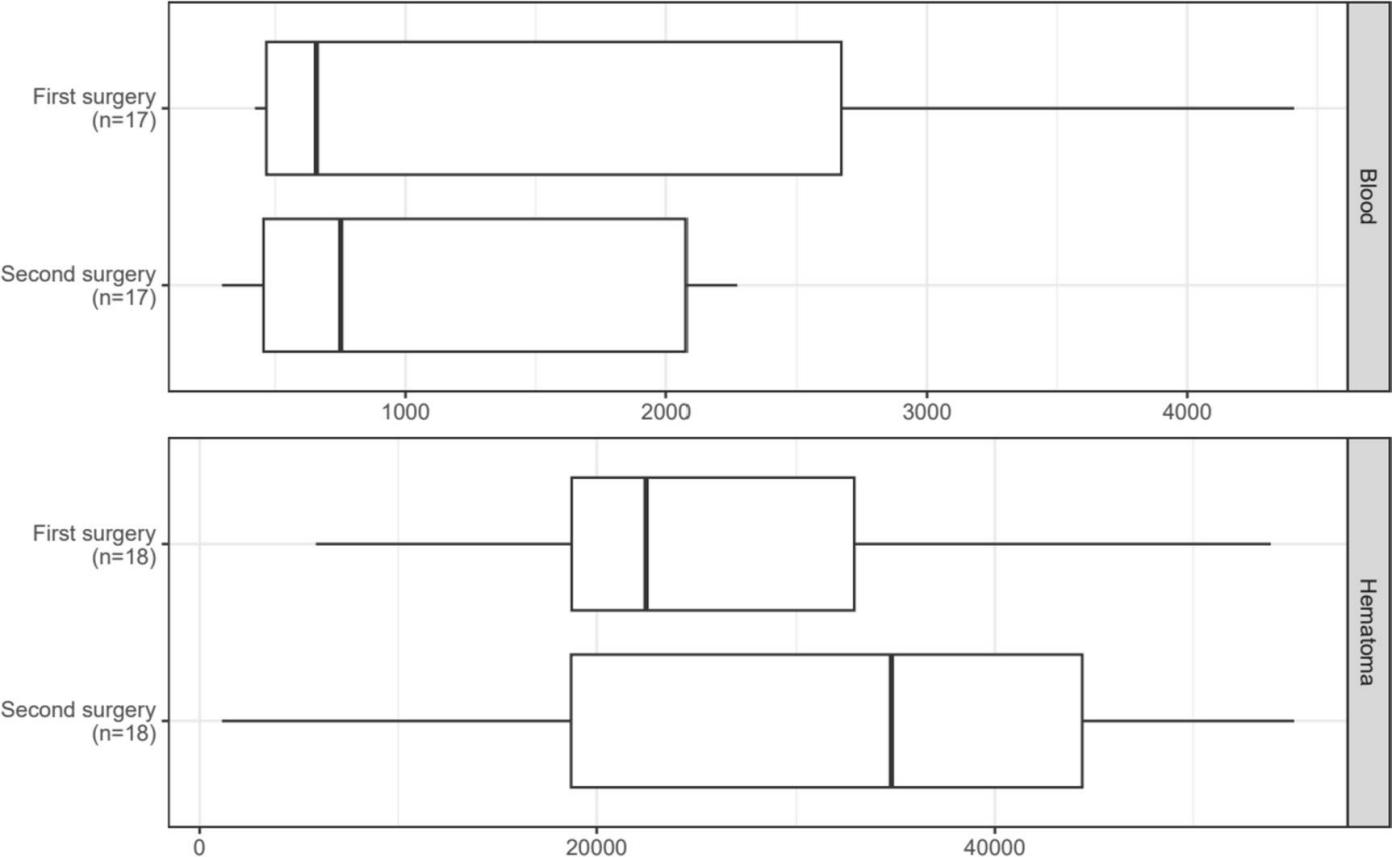
Boxplot presenting the comparison of the uPAR levels in systemic blood and hematoma membranes between the first and second operations in patients with recurrent CSDH. No differences were found between the first and second operations in systemic blood (*p* = 0.065) or hematoma fluid (*p* = 0.13)

To investigate a possible increase in the pathophysiological inflammation during a recurrent CSDH, we compared the levels of systemic and subdural uPAR between the first and second operation in patients with recurrent CSDH. We found no significant difference between uPAR levels in systemic blood between the first or second operation (first operation: 657 pg/L (465–2670) vs. second operation: 751 pg/L (454–2076), *p* = 0.065) or in hematoma fluid (first operation: 22,487 pg/L (18,740–32,915) vs. second operation: 34,786 pg/L (18,710–44,399), *p* = 0.13) (Fig. 5).

## Discussion

To our knowledge, this is the first study to identify uPAR in hematoma fluid, hematoma membrane, dura mater, or systemic blood from patients with CSDH. We found increased uPAR levels in the hematoma membrane compared to the dura mater and in the hematoma fluid compared to systemic blood, indicating a possible role of uPAR in CSDH pathophysiology. Furthermore, we found that dural uPAR levels could serve as a predictor of recurrent CSDH, but the number of cases examined with recurrent CSDH was low. To provide knowledge of the potential pathophysiological role of uPAR in the process of CSDH recurrence, uPAR levels in the hematoma fluid were compared between the first and second operations for patients with recurrent CSDH, but no significant difference was found.

### CSDH and uPAR

Detection of brain uPAR is not a novel entity, and uPAR has been extensively investigated in the pathophysiology of brain tumors, demonstrating a high uPAR level in malignant gliomas and metastases and a correlation between a high uPAR level and aggressive tumor activity and shorter patient survival [23–26]. uPAR is activated following a stress response and is strongly induced during inflammation [27]. The biomarker has been described in macrophages/microglia within the inflamed central nervous system (CNS) in Alzheimer’s disease, traumatic brain injury, multiple sclerosis, cerebral malaria, HIV, dementia, and Creutzfeldt–Jakob disease [28–34]. As an interesting observation, the systemic uPAR levels of CSDH patients were comparable to systemic uPAR blood levels in patients with head and neck squamous cell carcinomas, suggesting a comparable pro-inflammatory state between these two patient populations [25]. From an angiogenetic perspective, upregulation of uPAR increases vascular permeability by increasing the degradation of VE-cadherin, an endothelial-specific adhesion molecule located at the junction between endothelial cells, resulting in an increased bleeding probability [35, 36]. In fibrinolysis, uPAR is indirectly involved, as several members of the fibrinolytic system, including uPA, plasmin, and metalloproteases, are all able to cleave uPAR [37, 38]. This interacts with the adhesive and migrative functions of uPAR and may result in a uPAR-derived fragment that reacts with fibrinolytic processes, possibly leading to pathological disorders [39]. Correspondingly, uPAR levels in healthy CNS have been described as low or absent [40]. Our study supports the involvement of inflammatory, fibrinolytic, and/or angiogenetic cascades in CSDH pathophysiology, including a possible contribution of the plasminogen activation system.

### Molecular Risk Profile for CSDH Recurrence

The identification of a risk profile for recurrent CSDH holds substantial clinical potential, as it may lead to personalized treatment of the sub-cohort of CSDH patients in increased recurrence risk, while avoiding overtreatment of the entire CSDH population. Several studies on risk factors have been published, but the focus has largely been on patient characteristics [41, 42]. CSDH recurrence may be driven by a higher level of angiogenetic, fibrinolytic, and/or inflammatory involvement. As systemic anti-inflammatory treatment has proven successful in reducing CSDH recurrence, it is obvious that the involvement of specific inflammatory molecules may constitute a potential medical target [22]. This has been investigated in a few studies, but it still lacks substantial evidence [43, 44]. Our results indicate a possible correlation between immunohistochemical uPAR levels in the dura mater and the risk of recurrent CSDH. However, we experienced an immunohistochemical uPAR examination of the dura mater to be clinically less accessible, leaving this potential risk factor unsuitable for clinical use. Furthermore, we highlight that this result is peculiar, as uPAR levels were lower in the dura mater than in the hematoma membrane. Overall, this result is based on five samples and requires reexamination in a larger cohort.

### Clinical Implication

The significantly higher level of uPAR in the outer membrane found in this study, as well as the raised levels of uPAR in the hematoma fluid compared to peripheral blood, indicates that inflammation in CSDH is primarily a focal reaction at the site of the hematoma. This would explain the need for a relatively high dose of systemic anti-inflammatory medicine to reduce CSDH recurrence [22]. Systemic anti-inflammatory treatment for all CSDH patients is deemed impractical due to severe systemic side effects [22, 45]. However, as CSDH is presumably a disease primarily involving the subdural fluid and surrounding hematoma membrane, focal intracavity treatment to block angiogenetic, inflammatory, or fibrinolytic processes in CSDH development would likely reduce systemic side effects while inducing a strong targeted effect.

### Limitations

This study has several limitations. First, multiple factors may cause an inflammatory response, including hypertension, chronic alcohol abuse, hepatic diseases, renal insufficiency, diabetes mellitus, and several others [41, 42, 46–50]. These factors may theoretically elevate uPAR levels. We registered Charlson’s comorbidity index and found no difference between patients with and without recurrent CSDH; however, the inflammatory influence of other conditions may be a confounder.

Second, the immunohistochemical samples were generally limited, and especially from patients with recurrent CSDH, the number was only 5. In our department, if the recurrent CSDH is optimally localized with the *punctum maximum* of the hematoma beneath the previous burr hole from the primary CSDH operation, the burr hole is reused in the recurrent CSDH operation. This limits the possibility of collecting dura mater and hematoma membrane biopsies from patients with recurrent CSDH, which may reduce the implacability of these results. Therefore, the results should be regarded as exploratory only.

Finally, we found a statistical difference in the baseline characteristics of our study population between patients with and without recurrence. Bilateral CSDH is a known risk factor for recurrent CSDH, but to our knowledge, male sex is a risk factor for CSDH in the background population but not a risk factor for recurrent CSDH. As such, our skewed distribution may be a variability in outcome [41, 51]. Although the analysis of the prediction of CSDH recurrence may not be applicable to female CSDH patients, we would not expect a difference between male and female CSDH patients.

## Conclusion

We identified uPAR in the subdural fluid, hematoma membrane, dura mater, and systemic blood from patients with CSDH. The expression of uPAR is significantly higher in the subdural fluid and hematoma membrane compared to systemic blood and dura mater, indicating that the inflammatory mechanisms of CSDH are localized to the subdural fluid collection and surrounding hematoma membrane. This may have clinical implications for possible intracavity CSDH treatment.

### Supplementary Information

Below is the link to the electronic supplementary material.

Supplementary file1 (DOCX 14 KB)

## Data Availability

The datasets generated and/or analyzed during the study are available from the corresponding author on reasonable request.
