# Supplementary material for: Urokinase Plasminogen Activator Receptor: An Important Focal Player in Chronic Subdural Hematoma?
Source: Inflammation. 2024 Jan 18;47(3):1015–27. doi: 10.1007/s10753-023-01957-5 (PMC11147925; doi:10.1007/s10753-023-01957-5)
Supplement: Supplementary file 1 — Supplementary file1 (DOCX 14 KB) [file 10753_2023_1957_MOESM1_ESM.docx]

|  | **Non-recurrence, N** | **Median (IQR)** | **Recurrence, N** | **Median (IQR)** | **AUC (95%Cl)** |
| --- | --- | --- | --- | --- | --- |
| **Dura mater, histology** | **37** | 0.74 (0.26–1.93) | **5** | 1.65 (1.15–3.66) | 0.73 (0.57–0.89) |
| **Hematoma membrane, histology** | **28** | 17.04 (7.58–50.73) | **4** | 10.13 (7.16–15.05) | 0.62 (0.41–0.84) |
| **Systemic blood, Luminex ψ** | **114** | 933.47 (514.57–2333.67) | **27** | 797.42 (443.3–2670.47) | 0.5 (0.38–0.63) |
| **Hematoma fluid, Luminex** | **116** | 21318.58 (14355.25–30506.33) | **31** | 20482.98 (11631.72–25401.08) | 0.54 (0.43–0.66) |

**Supplementary Table 1.** Potential prediction of recurrent CSDH using uPAR levels in the dura mater*,* hematoma membrane, systemic blood, and hematoma fluid. Levels of uPAR in the dura mater significantly predicted the risk of recurrent CSDH. ψ The number of systemic blood samples is presented per CSDH case and not per patient. IQR: Interquartile range; AUC: Area under the curve
